# Does Feedback Seeking Help Safety Performance Improvement? The Role of Consideration of Future Consequence

**DOI:** 10.3389/fpsyg.2021.630669

**Published:** 2021-01-28

**Authors:** Tian-tian Zhang, Miao-miao Li

**Affiliations:** ^1^School of Economics and Management, University of Science and Technology Beijing, Beijing, China; ^2^School of Economics and Management, Beijing Information Science and Technology University, Beijing, China

**Keywords:** feedback seeking, feedback environment, safety performance, consideration of future consequence, three waves

## Abstract

The purpose of this paper is to examine how feedback seeking impact safety performance through feedback environment and the moderating role of consideration of future consequence. Correlation data were collected from 202 participants in three industries of China. Results indicate that feedback seeking is positively associated with feedback environment and safety performance, the feedback environment mediated the relationship between feedback seeking and safety performance. However, the positive effect of feedback environment on safety performance is more significant when consideration of future consequence is high. Overall, the findings highlight the critical importance of individual features in the research on safety performance. The conclusion is conducive to a more detailed understanding of the antecedents that affect safety performance and provides a new perspective for the improvement of safety performance.

## Introduction

Every year millions of employees suffer injuries and thousands even lose their lives at workplaces (Seo, 2005). Occupational injuries and accidents can have devastating consequences for both organizations and their employees. Good safety performance is the basis for personal health and development, and an important cornerstone for the stable development of the organization (Clarke, 2006). Griffin and Neal (2000) considered safety performance as individual work behavior associated with organizational safety, which is related to its psychological antecedents and can be systematically evaluated. Safety performance consists of two dimensions: safety compliance and safety participation. Safety compliance refers to some key safety activities that individuals must carry out to maintain work safety, such as abiding by safety regulations and wearing safety clothes (Wang et al., 2018). Safety participation refers to the voluntary participation in safety activities or safety meetings that are very meaningful for the improvement of organizational safety, in which individuals assume an additional volunteer role (Stride et al., 2013). Both safety compliance and safety participation are specific work behaviors, many studies have shown that employees with good safety compliance behaviors and safety participation behaviors can reduce accidents and injuries, improving safety performance. Although standards of technology and facility are believed to be key factors causing unsafe consequences, it is also recognized that equipment operation largely depends on individual behavior (Curcuruto et al., 2015). Therefore, it is necessary to study individual behaviors and their predictors that may influence safety performance. However, prior researchers have emphasized the role of macro factors such as the organization’s safety environment, safety atmosphere and organizational management commitment on safety performance and employee’s initiative have been neglected (Chen et al., 2017). Organizational safety is not the result of the organization acting alone, but the result of the interaction between individuals and organizations. The absence of individual perspective brings futile efforts, and both employees and leaders fall into the trap of “feedback vacuum” that employees can’t perceive leaders’ attitude even though leaders have made great efforts to safety problems. Therefore, it is necessary to examine the effect of individual initiative on safety performance. With safety environment has become more unpredictable, employees should actively improve their safety performance and guide their behaviors to meet organizational requirements and safety goals (Ginevra et al., 2016). It has long been considered that proactive behavior is critical for performance improvement and career development (Creed et al., 2009). As a kind of proactive behavior, feedback seeking was defined as an individual’s conscious acquisition of relevant information resources and commitment to determining the correctness and adequacy of relevant work behaviors to achieve a valuable final state (Lee and Kim, 2021). It includes employees actively acquiring useful information from their colleagues and supervisors to help individual’s clear key processes and reduce errors in the work (Ashford et al., 2018). Therefore, we examined the effect of feedback seeking on feedback environment.

The relationship between feedback seeking and safety performance is more likely a personal life story with internal and external interaction (Wehrle et al., 2019). Previous researches have explored the impact of external factors (environment) on the internal world (individuals), however, few have examined the backward path from the internal to external. We can’t truly understand the process of feedback if we ignored outer world changes triggered by individual initiative and behavior in the feedback loop. Feedback environment was defined as the informal feedback context between leaders and employees, coworkers and coworkers in the daily work environment (Prilop et al., 2019). The feedback environment goes beyond the feedback behavior itself, emphasizes environmental factors related to feedback and can affect the effect of feedback behavior, highlights the core of feedback usefulness, and solves the problem that it is difficult to effectively improve performance and employee self-development only by focusing on the feedback behavior (Dahling et al., 2017). Gong (2018) found that “What feedback environment lacks in feedback research makes up for emphasizing feedback’s multi-dimensional complexity and the effective construction of feedback receiver, encouraging feedback seeking. Compared with feedback, feedback environment can lead to more consistent expected results.” The more individuals engaged in feedback seeking behaviors, the more useful feedback and information they will receive. Thus, they will be more identified with their leaders and a supportive environment is naturally constructed, which will then promote their work development, such as safety performance (Whitaker and Levy, 2012). Here, we tested the indirect effect of feedback seeking on safety performance through feedback environment.

A constructive feedback environment promotes communication and feedback, which can help the individual obtain more valuable and useful information resources and help (Dahling et al., 2017). However, in practice, individuals in a similar feedback environment still show different safety performance. To understand individual behavior, it is necessary to understand the boundary conditions perceived by their own (Joireman et al., 2012). The connection between an individual’s internal characteristics and behavior might be weakened by the remote consequence (Joireman et al., 2006). Consideration of future consequence refers to the extent to which the individual considers the potential future outcomes of the current behavior and the extent to which the individual is affected by those potential future outcomes (Cao and Xia, 2020). Consideration of future consequence can effectively predict a lot of individual behaviors (Lewis et al., 2018), such as following rules and regulations, self-control, reducing alcohol abuse, etc. Safety performance can largely be considered as a future variable due to the lag between safety behavior and safety consequence. It is assumed that high consideration of future consequence is mainly concerned with the future rather than the immediate consequences of their actions. Moreover, similar patterns have been confirmed in other domains (Strathman et al., 1994). Therefore, we tested the moderating role of Consideration of future consequence in the relationship between feedback environment and safety performance.

**FIGURE 1:**
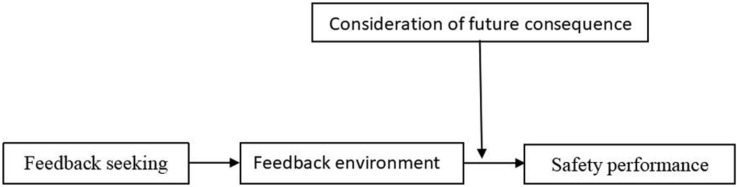
Test model.

Accordingly, we explored the indirect effect of feedback seeking on safety performance via feedback environment. Besides, we predicted consideration of future consequence to moderate the relationship between feedback environment and safety performance such that the positive relationship is stronger for employees with high consideration of future consequence than for low consideration of future consequence. Therefore, Figure 1 below showed the test model.

## Theory and Hypotheses

Ashford and Cummings (1985) defined feedback seeking as an individual’s conscious acquisition of relevant information resources and commitment to determining the correctness and adequacy of relevant work behaviors to achieve a valuable final state. Individuals who are likely to communicate with their leaders are better equipped to understand organizational policies and procedures (Ashford, 2003). Thus feedback seeking as employees’ initiative behavior will positively affect safety performance.

On the one hand, increasing feedback seeking behavior can improve employees’ sense of identity and belonging to the organization, and then their negative behaviors will be reduced. On the other hand, employees could obtain more chances to improve their working ability by seeking feedback (Anseel et al., 2015). At the same time, feedback behaviors provide individuals an opportunity to have access to supervisors and colleagues’ attitudes toward their performance, which can help them to adapt to an unpredictable work environment (VandeWalle et al., 2000). Even information from the same source can give individuals different perspectives on their behaviors, thus inducing behavioral changes and generating more positive organizational behaviors, which is beneficial to the improvement of safety performance. Thus, we formed the following hypothesis:

Hypothesis 1: Feedback seeking positively affects safety performance

Employee proactive behavior could be perceived by supervisors, who will then in turn provide them with more useful and reliable information and feedback (Rosen et al., 2006). As both a capacity and behavior, feedback seeking is derived from and also influences feedback environment. The valuable and reliable information in the feedback will promote employees’ performance and motivate leaders to develop a constructive feedback environment. Therefore, feedback seeking behavior will be further improved (Whitaker et al., 2016). Accordingly, we make the following hypothesis:

Hypothesis 2: feedback seeking positively effect on feedback environment

Feedback environment referred to the informal feedback context between leaders and employees, coworkers and coworkers in the daily work environment. It goes beyond feedback behavior itself and emphasizes environmental factors that can affect behavior effectiveness. Because negative feedback will damage individual images and reduce their self-esteem, individuals who care about seeking feedback don’t ask for it either under an informal circumstance. Even supervisors pay attention to give feedback but have little effect, so shaping a supportive feedback environment is of great significance (Chen et al., 2018). In a constructive feedback environment, employees are given more reliable and accurate information and individuals are also encouraged to have more feedback seeking behavior (Dahling et al., 2017). This process is important to improve safety performance because a constructive feedback environment provides individuals with more chances to acquire feedback and achieve the objective of the feedback’s content. Constructive feedback help employees obtain useful and valuable information to learn, develop, and improve their safety performance (Rosen et al., 2006). Both incentive and supportive feedback environment will make employees feel respected and concerned (Cutumisu and Lou, 2020). It drives individuals to have more positive experiences and generate more positive organizational behaviors; safety performance is naturally being improved. Employees in the supportive feedback environment are more likely to receive high-quality feedback to improve role clarity, job satisfaction and performance (Cutumisu, 2019). Accordingly, we make the following assumption:

Hypothesis 3: Feedback environment mediate the effect of feedback seeking on safety performance

Consideration of future consequence is the extent to which the individual considers the potential future outcomes of the current behavior and the extent to which the individual is affected by those potential future outcomes, and it predicts safety compliance, safety participation (Joireman et al., 2006). Individuals with high consideration of future consequence pay more attention to the future consequences of behavior, and very little attention to the immediate consequences of action (Davis, 1983). This will directly affect information selection and utilization in the feedback environment. According to construal level theory (Liberman et al., 2007), they will pay more attention to the long-term goals, thus they will extract, select and regroup information in the feedback environment that is constructive to solve problems at present and their behavior will be less aggressive. Individuals with high consideration of future consequence seeking feedback in the environment is a performance-oriented action that makes leaders concern about workplace issues that may threaten safety performance (Jiang, 2016). In this situation, employees will choose key feedback information in the feedback environment consciously, which will strengthen the positive effect between feedback environment and safety performance.

In the contrary, individuals with low consideration of future consequence highly valued the immediate consequences of behavior, and the delayed consequences of behavior have become less of a focus (Joireman et al., 2012). They prefer actions that can bring immediate profit, thus they are more likely to break down rules and regulations to acquire immediate profit even though that will threaten safety performance. Under this context, although feedback environment provides employees with valuable information and feedback, they will filter and ignore that consciously to meet their own needs (Rebetez et al., 2016). Therefore, we formed the following hypothesis:

Hypothesis 4: Consideration of future consequence moderate the effect between feedback environment and safety performance

## Method

### Participants and Procedures

In this study, a total of 325 surveys were distributed and 202 valid questionnaires (completed at all phases of the study and did not have invalid answer such as only write one score in the whole questionnaire, write the order score, et al.) were received (efficiency response rate of 62%). Participants were full-time employees from 3 different firms in China. During the investigation, the participants were told that the investigation was anonymous and did not involve any interests, only for academic research to better understand the characteristics of the work, and there was no right or wrong answer. And we use three phrases to complete this research, with an interval of 3 months. First, employees responded to feedback seeking and demographic variable measures. Second, employees completed feedback environment measures and consideration of future consequence measures. Finally, we assessed employees’ safety performance. Participants were asked to write their word identification numbers on each measure so their answers could be matched across the three phrases.

Among the participants, 61.2% (*n* = 124) are males and 38.8% (*n* = 78) are female. Their average age was 35.6 years, with 34.2% (*n* = 69) aged 20–30 years, 54.5% (*n* = 110) aged 31–40 years, and 11.3% (*n* = 23) aged 41 years or more. Regarding to the tenure, their average professional experience was 4.8 years, 52.9% (*n* = 107) had worked for less than 5 years, 20.8% (*n* = 42) had worked for 6–10 years, and 26.3% (*n* = 53) had worked for more than 10 years. In terms of education level, 32.3% (*n* = 65) had obtained a junior college degree or less, 54.4% (*n* = 110) were college graduates, 13.3% (*n* = 27) had a master’s or doctoral degree. In our sample, 19.4% (*n* = 39) are security police, 18.1% (*n* = 36) are special police, 17.6% (*n* = 36) are criminal police, 28.5% (*n* = 58) are other types of police, such as Internet police. Our samples are well representative.

### Measures

#### Feedback Seeking

We adopted the feedback seeking scale proposed by Morrison (1993), which taps into feedback inquiry and feedback monitoring. This scale contains 11 items, item includes “By observing my leaders’ response to my work, I can find out if he is satisfied with my work.” All variables were measured on a seven point Likert scale from 1 (very inconsistent) to 7 (very consistent). Because we want to measure feedback seeking as a complete variable, in this research we used the average of all items to represent the score of feedback seeking. The Cronbach’s alpha for feedback seeking was 0.93.

#### Feedback Environment

We measured the feedback environment with a scale consisting of 21 questions compiled by Steelman et al. (2016). Items like “My leader knows my performance very well” and so on. This scale is Likert seven point scale from 1 (very inconsistent) to 7 (very consistent). This Likert scale assesses seven dimensions including source credibility, feedback quality, feedback delivery, the accuracy of favorable feedback, the accuracy of unfavorable feedback, source availability, and promoting feedback seeking. The Cronbach’s alpha for feedback environment was 0.94.

#### Safety Performance

Safety performance was measured by a four items scale developed by Neal et al. (2000), which assesses two dimensions of safety performance: safety compliance and safety participation. Item includes: “A lot of safety policies and procedures are not practical when they work.” This scale is seven point Likert scale from 1 (very inconsistent) to 7 (very consistent). The Cronbach’s alpha for feedback seeking was 0.85.

#### Consideration of Future Consequence

We used Probst, Graso, Estrada’s (1994) scales with six items to measure this variable. Items include “I think about what the future holds and influence it with my daily actions.” All the questions were asked on a seven point Likert scale from 1 (very inconsistent) to 7 (very consistent). The Cronbach’s alpha for feedback seeking was 0.93.

#### Control Variable

Demographic information about gender, age, police category, job tenure, and education are control variables.

## Results

### Correlation Analysis and Confirmatory Factor Analysis

Table 1 shows that feedback seeking has a positive relationship with feedback environment, consideration of future safety consequence and safety performance. The feedback environment is significantly positively correlated with consideration of future consequence and safety performance. Consideration of future consequence positively affects safety performance.

**TABLE 1:** Means, standard deviations, and correlations of all measures.

Measure	*M*	*SD*	1	2	3	4
1. Feedback seeking	5.55	1.20	–			
2. Feedback environment	5.31	1.27	0.82**	–		
3 Safety performance	5.22	0.97	0.69**	0.65**	–	
4 Consideration of future consequence	5.21	0.94	0.72**	0.76**	0.76**	–

n = 202; ** *p* < 0.01.

In this study, we employed structural equation model (SEM) to conduct the discrimination validity of confirmatory factor analysis (CFA) using AMOS 21.0. A reasonable model fit is indicated when the CFI and IFI are above 0.90 and the RMSEA is below 0.08. This study compared a 4-factor model with two 3-factor models, a 2-factor model and a single-factor model. As is shown in Table 2, the indicators of the 4-factor model are superior to others, indicating that the 4 variables have good discriminant validity.

**TABLE 2:** Confirmatory factor analysis of discrimination validity.

Model	Factor load	χ^2^/*df*	*GFI*	*RMSEA*	*CFI*	*NFI*	*IFI*
Model 1	4 factors: FS, FE, SP, CFC	2.57	0.98	0.09	0.95	0.95	0.96
Model 2	3 factors: FS, FE, CFC + SP	2.75	0.49	0.12	0.73	0.64	0.74
Model 3	2 factors: FS, FE + CFC + SP	2.96	0.44	0.12	0.69	0.60	0.69
Model 4	1 factor: FS + FE + SP + CFC	3.36	0.36	0.13	0.64	0.56	0.64

*FE, feedback environment; FS, feedback seeking; SP, safety performance; CFC, consideration of future consequence.*

*“+” = two factors combined into one factor.*

### The Mediating Role of Feedback Environment

As shown in Table 3, after controlling for the effect of demographic variables, model 2 validated the direct effects of feedback seeking on safety performance, and hypothesis 1 was supported. Model 1 better illustrates the direct impact of feedback seeking on the feedback environment, hypothesis 2 was supported. Model 3 showed that feedback environment has a significant impact on safety performance. After adding the variable of feedback environment, feedback seeking has a significant effect on safety performance, but the direct effect is smaller, indicating that feedback environment can partially explain safety performance. We adopted the SPSS PROCESS to calculate the indirect effects. The results in Table 3 showed that the indirect effect of feedback seeking on safety performance via feedback environment was significant, with the confidence interval was [0.27, 0.52], suggesting that the indirect effect was significant. Taken together, these results supported hypotheses 1, 2, and 3.

**TABLE 3:** Hierarchical regressions results about mediation and moderation effect.

Measures	Model 1	Model 2	Model 3	Model 4
		
	Feedback seeking	Safety performance		
Feedback seeking	0.80**	0.76**	0.39**	0.23**
Feedback environment			0.46*	0.64**
Consideration of future consequence				0.75**
Feedback seeking* Consideration of future consequence				0.06**
Intercept	1.52**	1.28**	0.59	−1.69**
*R*^2^	0.70	0.66	0.73	0.78
Adjust *R*^2^	0.68	0.64	0.71	0.76
*F*	40.72**	34.35**	40.62**	42.69**

n = 202; * *p* < 0.05, ** *p* < 0.01;

### The Moderating Role of Consideration of Future Consequence

Results in Table 3 confirm that the relationship between the interaction terms of the feedback environment and consideration of future consequence on safety performance is significant. Then, we obtained the conditional indirect effect under different values of the moderating variable. As can be seen from the results in the left part of Table 4, when consideration of future consequence is poor, the effect of feedback environment on safety performance is −0.02 (CI [−0.33, 0.31]). When consideration of future consequence is good, the effect of feedback environment on safety performance is 0.26 (CI [0.05, 0.48]). The results show that the effect of feedback environment on safety performance is significant only when consideration of future consequence is good. The right side of Table 4 shows that the moderated mediation effect judgment index is 0.10 (CI [0.01, 0.26]), a confidence interval does not contain 0. Hypothesis 4 is supported.

**TABLE 4:** Results for a conditional direct effect of feedback environment on safety performance across levels of considerations of future consequence.

Variable		Conditional indirect effect				moderated mediation effects			
		Effect	SE	LL95%CI	UL95% CI	Index	SE	LL	UL
Consideration of future consequence	Low (M−1SD)	−0.02	0.16	−0.33	0.31	0.10	0.06	0.01	0.26
	High (M+1SD)	0.26	0.11	0.05	0.48				

*n = 202.*

*LL, lower limit; CI, confidence interval; UL, upper limit.*

**FIGURE 2:**
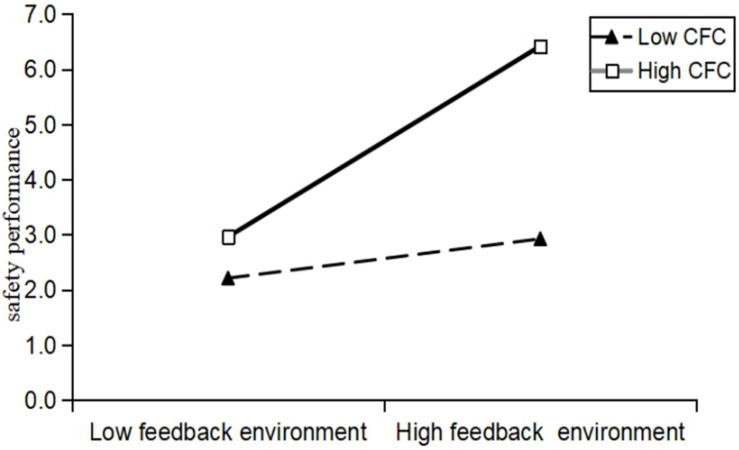
The moderating effect of consideration of future consequence on the relationship between feedback environment and safety performance.

We plotted the relationship between feedback environment and safety performance when moderating effects of consideration of future consequence at different levels. As seen in Figure 2, we can see that when consideration of future consequence is poor, the effect of feedback environment on safety performance is relatively small, which is a relatively flat curve. Under the condition that consideration of future consequence is high, the effect of feedback environment on safety performance is significant, which is a steep curve with a higher slope. In conclusion, hypothesis 4 was supported.

## Conclusion and Discussion

The above results support our assumptions: the feedback environment plays a mediating role in the relationship between feedback seeking and safety performance and consideration of future consequence moderates the relationship between feedback environment and safety performance. The research shows that when employees have a high consideration of future consequence, the feedback environment has a more significant impact on safety performance. We have made contributions to the literature of safety performance by highlighting the importance of consideration of future consequence as a moderator.

### Theory Implication

First, our work is the first empirical research on the relationship between feedback seeking and safety performance and thus provides a new perspective for improving employee safety performance. At the same time, it also makes up the defect that previous studies ignore the motivation behind seeking feedback seeking and then lead to inconsistent conclusions of the relationship between feedback seeking and performance.

Second, it enriches the research of feedback seeking causal reversibility. It is confirmed that more frequent feedback seeking based on poor job performance might eventually contribute to improving performance. However, we think some other factors may follow the same logic. We explored that feedback seeking has a positive effect on safety performance through feedback environment, which was similar to the dynamic, reciprocal model of feedback seeking. Previous researches have shown that the feedback environment has an effect on feedback seeking but has ignored the reverse path possibility. This study provides a new angle on the relationship between feedback seeking and feedback environment, the research perspective of antecedent variables related to the feedback environment has been changed.

Third, We combine macro factors with micro perspectives to explore the influence mechanism of safety performance. The performance was not only determined by individuals or the environment but the interaction of the two. It solves the difficult problem of the implementation of organizational policies caused by the past literature that only focused on the unilateral factors of macro or micro.

Finally, this study expands the application of construal level theory. Mashi et al. (2018) discussed the problems in the field of human resource security by introducing the variable of consideration of future consequence and combining it with the construal level theory. This study defines that the variable of consideration of future consequence is the boundary condition of the impact of feedback environment on safety performance, and solves the problem of inconsistent performance of individuals in the same feedback environment. It provides a new perspective and theoretical support for us to study the differences of organizational psychological characteristics and behaviors.

### Practical Implication

Concerning practical implications. First, leaders should not only pay attention to the construction of organizational safety policies and systems but also actively promote communication. Leaders should encourage employees to seek feedback and help them improve their feedback seeking. A complete and smooth communication channel is the basic guarantee for individuals’ development.

Secondly, leaders should encourage individuals to actively participate in activities related to safety to build a positive safety atmosphere in the organization. And leaders should be aware of factors that may threaten working environments. A constructive and positive environment leading to more organizational citizen behaviors.

Finally, leaders should realize employees’ different characteristics. Adopting approaches that are more suitable for employees will acquire better promote employee performance and facilitate their career development.

### Limitations and Future Research

Although this study has made a lot of contributions in practice and theory, it still has some limitations. Firstly, the data adopted in this study are cross-sectional data. The safety performance may be lagging, so our measurement of safety performance may be biased. Therefore, follow-up investigation can be considered in the future, and a comprehensive self-report scale can be used to accurately and comprehensively obtain the situation of safety performance.

Secondly, this study has adopted a variable-centered approach, assessing how a composite score of seven feedback environment facets relates to creative performance. This assumes that employees have similar perceptions across all facets, ignoring the possibility that different profiles, or constellations, of coworker feedback environment perceptions may exist. Future research need to seek to address this limitation by adopting a person-centered research approach to identify profiles of feedback environment perceptions, and test the relations of these profiles with important feedback environment criteria.

Finally, this study explores the ways to influence safety performance from the perspective of feedback seeking. In the context of this study, feedback seeking is an active behavior driven by internal motivation. But the research on motivation shows that the intrinsic motivation and extrinsic motivation of individuals can be divided into many different motivations according to different purposes. For example confirmation motivation, intake motivation, differentiation motivation, etc. Future studies can explore different impacts of various motivation-driven feedback seeking on safety behaviors, which can help organizations more accurately meet the different needs of individuals and achieve a satisfactory performance level.

## Data Availability Statement

The original contributions presented in the study are included in the article/supplementary material, further inquiries can be directed to the corresponding author/s.

## Ethics Statement

This study is reviewed and approved by American Psychological Association (APA) Ethics Committee Rules and Procedures, APA Ethics Committee with written informed consent from all participants. All participants have given written informed consent following the Declaration of Helsinki. This study was also approved by the Ethics Committee of School of Economics and Management, University of Science and Technology Beijing. The patients/participants provided their written informed consent to participate in this study. Written informed consent was obtained from the individual(s) for the publication of any potentially identifiable images or data included in this article.

## Author Contributions

Both authors contributed to the article and approved the submitted version.

## Conflict of Interest

The authors declare that the research was conducted in the absence of any commercial or financial relationships that could be construed as a potential conflict of interest.
